# Tools for integrated sequence-structure analysis with UCSF Chimera

**DOI:** 10.1186/1471-2105-7-339

**Published:** 2006-07-12

**Authors:** Elaine C Meng, Eric F Pettersen, Gregory S Couch, Conrad C Huang, Thomas E Ferrin

**Affiliations:** 1 Computer Graphics Laboratory, University of California San Francisco, 600 16th Street, San Francisco, CA 94143-2440, USA

## Abstract

**Background:**

Comparing related structures and viewing the structures in the context of sequence alignments are important tasks in protein structure-function research. While many programs exist for individual aspects of such work, there is a need for interactive visualization tools that: (a) provide a deep integration of sequence and structure, far beyond mapping where a sequence region falls in the structure and vice versa; (b) facilitate changing data of one type based on the other (for example, using only sequence-conserved residues to match structures, or adjusting a sequence alignment based on spatial fit); (c) can be used with a researcher's own data, including arbitrary sequence alignments and annotations, closely or distantly related sets of proteins, etc.; and (d) interoperate with each other and with a full complement of molecular graphics features. We describe enhancements to UCSF Chimera to achieve these goals.

**Results:**

The molecular graphics program UCSF Chimera includes a suite of tools for interactive analyses of sequences and structures. Structures automatically associate with sequences in imported alignments, allowing many kinds of crosstalk. A novel method is provided to superimpose structures in the absence of a pre-existing sequence alignment. The method uses both sequence and secondary structure, and can match even structures with very low sequence identity. Another tool constructs structure-based sequence alignments from superpositions of two or more proteins. Chimera is designed to be extensible, and mechanisms for incorporating user-specific data without Chimera code development are also provided.

**Conclusion:**

The tools described here apply to many problems involving comparison and analysis of protein structures and their sequences. Chimera includes complete documentation and is intended for use by a wide range of scientists, not just those in the computational disciplines. UCSF Chimera is free for non-commercial use and is available for Microsoft Windows, Apple Mac OS X, Linux, and other platforms from .

## Background

Integration of protein sequence and structure information is essential in many problem domains, including structural biology, protein engineering, and drug design. A suite of tools within UCSF Chimera [1] has been developed for studying sequence-structure relationships and comparing related structures.

Common tasks in sequence-structure work include: (A) displaying information from a sequence alignment on one or more corresponding structures, or displaying information from the structures on the alignment; (B) superimposing structures so that they can be compared; (C) generating a structure-based sequence alignment.

The Multalign Viewer extension of Chimera displays sequence alignments and automatically associates structures with sequences in the alignment. Structures can be superimposed using the alignment, and sequence-related data such as conservation can be shown on the structures. If one does not already have a sequence alignment, the MatchMaker extension can be used to generate sequence alignments and superimpose structures accordingly. A novel score incorporating both secondary structure and residue type is used to align the sequences. Completing the cycle, the Match -> Align extension constructs sequence alignments from pre-existing superpositions of structures.

These tools work together within Chimera to enhance the understanding of sequence information in the context of structure and vice versa. Below, we describe the tools in more detail and discuss their advantages and disadvantages relative to other programs.

## Implementation

The implementation of the Chimera system is described elsewhere [1]. The tools described in this paper (Multalign Viewer, MatchMaker, Match -> Align) are all implemented as extensions to Chimera and are distributed along with Chimera. They are written in the Python scripting language and their user interfaces are implemented using Tkinter, Python's interface to the Tk GUI toolkit. Chimera's normal extension mechanisms are used to make the tools available in Chimera's "Tools" menu and to register file types that the tools can open, which then appear in the list of types in Chimera's main file-opening dialog.

The Multalign Viewer, MatchMaker, and Match -> Align tools are accessed from the Structure Comparison section of the Tools menu. Descriptions of parameters refer to the default settings in Chimera version 1.2199.

## Results and discussion

### Multalign Viewer

Sequence alignments in several common formats (Clustal ALN, aligned FASTA, GCG MSF, GCG RSF, aligned NBRF/PIR, and Stockholm) can be opened in Chimera and shown with Multalign Viewer. When a sequence alignment and a structure have been opened in Chimera (in either order), the sequence of the structure is compared to each of the sequences in the alignment. The structure is then ***associated ***with the alignment sequence that yields the fewest mismatches, if within a user-specified tolerance. The default mismatch tolerance is 1/10 the number of residues in the structure chain. Reasons for mismatches include point mutations, portions of a structure missing due to insufficient density for coordinates to be determined, and association with a homologous protein rather than the same protein (a useful sequence alignment might not include the sequence of the structure of interest, or even any sequence for which a structure is known). For rapid automatic association, it is assumed that gaps in the structure sequence relative to the alignment sequence can only occur where residues are missing from the structure. Multiple structures can be associated with multiple sequences, or even with the same sequence. When a sequence is associated with a structure, its name is shown in bold over a rectangle of the structure's default color (or if the sequence is associated with multiple structures, a dashed outline).

Sequence-structure associations can be changed or added if the automatic procedure does not give the desired result. If an association did not meet the criteria for the automated approach but is requested by the user, the Needleman-Wunsch algorithm [2] with identity scoring is used to align the structure sequence with the alignment sequence. Although slower than the automatic-association method, this approach will yield the optimal alignment even in those rare cases where automatic association fails (such as when the alignment sequence has an internal gap where the structure sequence does not).

If the sequence names are based on PDB identifiers [3] (for example, in alignments downloaded from HOMSTRAD [4]), the structures can be fetched over the internet from the PDB and opened automatically, instead of explicitly by the user. Similarly, if the sequence names are based on SCOP [5,6] domain identifiers, the domain structures can be fetched from ASTRAL [7] and opened automatically.

Association permits many types of sequence-structure crosstalk, including the following: selections made with the mouse in the sequence are highlighted in the structure, and vice versa; the structure residue number is displayed when the cursor is placed over a residue in the sequence; and structures can be matched based on the sequence alignment. By default, structure matching uses the alpha-carbon pairs corresponding to every column in the alignment (where neither associated sequence has a gap) in a least-squares fit. Alternatives include using only columns highlighted by the user, or columns with high conservation. The fit can be improved iteratively by pruning alpha-carbon pairs that are not well superimposed. In each cycle, the atom pairs removed are either the 10% farthest apart of all pairs or the 50% farthest apart of all pairs exceeding a specified cutoff distance, whichever is fewer. Iteration allows the best-matching regions to govern the fit and conformationally dissimilar regions like flexible loops to be excluded, even though they may be aligned in the sequence alignment. While this matching is inherently pairwise, a multiple superposition can be achieved by using a consistent reference structure, for example, by matching B to A and C to A. Match statistics are reported in the Chimera Reply Log, which can be opened from the Favorites menu. The matched structures can be saved as PDB or Mol2 files.

A unique feature of Multalign Viewer is the treatment of ***headers***, annotations that appear above the sequences in an alignment. Header information is automatically propagated as a residue ***attribute ***to any associated structures. If the header is numerical (shown above the alignment as a histogram), associated structures can be colored by the attribute value and/or shown as a "worm" with thickness scaled by the attribute value, using Chimera's Render by Attribute extension.

**Figure 1:**
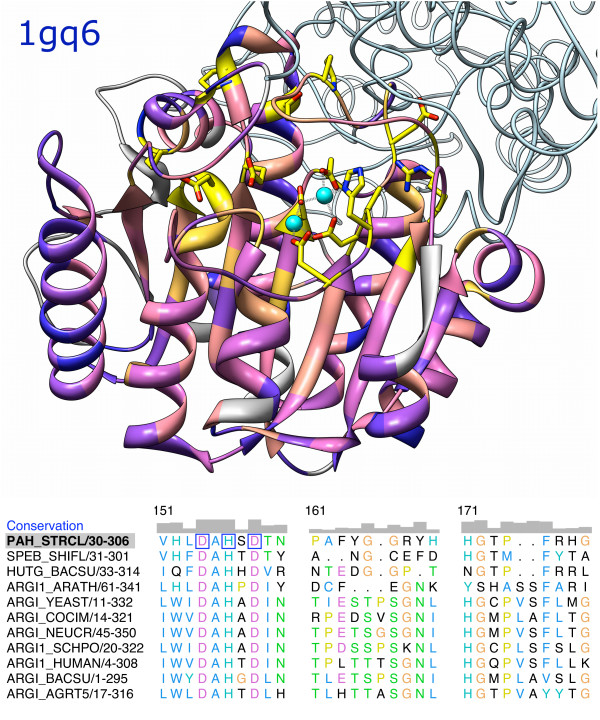
**Protein structure and associated sequence alignment**. The structure of proclavaminate amidino hydrolase [PDB:1gq6] and the seed alignment from Pfam [33] for its family. Only a portion of the alignment is depicted. One chain of the trimer, shown as a ribbon, is associated with the first sequence in the alignment. The other two chains are shown as light blue backbone traces. The ribbon is colored from blue to pink to yellow with increasing conservation. Gray ribbon segments represent residues in columns for which conservation was not calculated due to a high proportion of gaps (0.5 or higher). Conservation values, also shown as histogram bars above the alignment, were obtained with the entropy-based measure in AL2CO [8] and "independent counts" weighting. Active site ions are shown in cyan. Residues in the structure that are completely conserved in the alignment have their side chains displayed and are indicated with blue boxes on the alignment. The part of the alignment that is shown includes conserved (boxed) ion-binding residues in a loop to the lower right of the ions. Highly conserved residues are primarily involved in the active site or inter-subunit interactions. Residues in the protein core are moderately conserved. In the sequence alignment, Clustal X coloring is used for the residue one-letter codes. The alignment part of the figure was exported directly from Multalign Viewer as Encapsulated PostScript.

Figure 1 shows a structure colored by the values in the Conservation header. This header is present by default. Sophisticated methods for calculating conservation are provided by the AL2CO program of Pei and Grishin [8]: entropy-based, variance-based, or sum of pairs, with or without sequence weighting. Any number of additional headers can be defined arbitrarily by reading in a simple text file. Existing headers, including Conservation, can be saved into the same type of text file. After a header is loaded, it is displayed above the alignment (although it can be hidden if desired) and is available as an attribute of residues in any associated structures. Header information that consists of only a single character or digit per column could also be read in as part of an alignment in Stockholm format. The header file format, however, allows use of multiple-digit numbers: values ranging from large negative to large positive are transformed for histogram display (and transformed back into the original numbers if written to a file). The header mechanism allows user-calculated values to be incorporated into an alignment and visualized on structures without Chimera code development.

The font size, text wrapping, and residue letter coloring can be adjusted in Multalign Viewer's preferences. Minor editing can be performed. Alignments or parts of alignments can be written out in any of the formats that can be read, with optional removal of all-gap columns. The sequence window contents can also be saved as an Encapsulated PostScript file for publication.

There are many additional features, including searching for a particular sub-sequence or PROSITE [9] pattern, display of secondary structure elements on the alignment, calculation of pairwise percent identities, and superposition assessment by generating a "closeness of fit" residue attribute. The state of Multalign Viewer is included in saved Chimera sessions. A user can save a session, exit from Chimera, and later resume work after restoring the session in Chimera.

### MatchMaker

Most structural comparisons require the structures to be superimposed in some sensible way. A user may have a collection of structures to be compared, but no pre-existing sequence alignment to govern matching. The MatchMaker extension of Chimera constructs pairwise sequence alignments and uses them to superimpose the structures. The sequence alignment(s) can be kept hidden or opened in Multalign Viewer. As in structure matching with Multalign Viewer, the fit can be improved iteratively by pruning residue pairs far apart in space, match statistics are reported in the Chimera Reply Log, and a multiple superposition can be achieved by using a consistent reference structure. MatchMaker can be run from its graphical interface or from the Chimera command line.

The standard Needleman-Wunsch [2] and Smith-Waterman [10] algorithms are available for producing global and local sequence alignments, respectively. The chains to match can be specified explicitly, or MatchMaker can identify the best-matching chains based on alignment scores. Alignment scores can include residue similarity, secondary structure information, and gap penalties:

#### Residue similarity

Any of several common substitution matrices (BLOSUM [11] or PAM [12]) can be used.

#### Secondary structure

This contribution is analogous to residue similarity, but instead of residue types, the values depend on what type of secondary structure the residues fall within: helix, strand, or other.

#### Gap penalties

Alignment scores can be penalized for opening and extending gaps. When secondary structure scoring is used, there can be different penalties for opening gaps within different types of secondary structure.

The default settings are to use the Needleman-Wunsch algorithm with BLOSUM-62 and 30% weighting of the secondary structure term (thus 70% weighting of the residue similarity term). These settings perform well in many situations, but users can easily adjust these parameters, as well as the gap penalties and secondary structure matrix values, via the graphical interface. Secondary structure scoring can be turned off or its weight adjusted with a slider. Without secondary structure scoring, the method collapses to purely standard protocols for sequence alignment and scoring.

MatchMaker originally did not use secondary structure information. However, we often evaluated its performance by showing secondary structure on the sequence alignment, begging the question of why such information was not used directly. Using secondary structure extends the applicability of MatchMaker to more distantly related proteins, where purely sequence-based methods either cannot achieve a correct alignment, or can achieve a correct alignment, but only with a narrow set of parameters that may be hard to identify.

For example, enolase [PDB:4enl] and mandelate racemase [PDB:2mnr] are homologous but share less than 20% identity; common features include three metal-binding residues in the active site [13]. MatchMaker with default settings superimposes the structures correctly. In the sequence alignment, two of the three metal-binding residues are paired correctly and the third is offset by one position. Except for slight changes in the offset of the third metal-binding residue, this result is robust to changes in the weight of the secondary structure term (5–100%) or switches to other matrices, except PAM-40. Without secondary structure scoring, only the BLOSUM-35–45 matrices yield roughly correct superpositions, but with the active site residues poorly superimposed in space and incorrectly paired in the sequence alignments. Data for additional pairs are presented below, in the section on matching distantly related proteins.

Fit iteration does not change the sequence alignment; it just prunes columns of the alignment from the least-squares fit of the structures. Thus, the requirement for success with MatchMaker is merely that enough columns in the sequence alignment are structurally correct to dominate the initial superposition. Pruning during iteration will then exclude the "wrong" positions, yielding a correct superposition based on fitting the remaining pairs. Because relatively few pairs may remain at this stage, MatchMaker alone does not yield a full set of residue equivalences between structures. Residue equivalences can be obtained using Match -> Align, described next.

### Match -> Align

Given a superimposed set of two or more protein structures, Match -> Align constructs a corresponding sequence alignment. It does not matter how the input superposition was generated; it could have been created interactively, or with a tool in Chimera such as MatchMaker, or with some other program. Residue types are not used, only the spatial proximities of alpha-carbons. The user specifies a cutoff distance and a column inclusion criterion: whether a residue must be within the cutoff distance of at least one other residue in the column or all other residues in the column. Structures related by circular permutation can be accommodated with sequence doubling. Match -> Align determines if sequence doubling is needed and if so, which sequence(s) should be doubled.

In the pairwise case, a dynamic programming algorithm is used to determine the sequence alignment that best represents the structural alignment. The score for aligning a pair of residues is (cutoff – distance) for distances no greater than the cutoff and -1 for distances greater than the cutoff. A gap penalty of zero is used so that spatial proximity overrules adjacency in sequence.

In the case of more than two structures, heuristics are required to render the problem tractable. For each pair of chains, all residue-residue pairings or "links" within the cutoff distance are identified. Links that cross when the sequences are laid out in parallel are removed, starting with the link with the most crossings, until no links between the pair cross. Alignment columns are then assembled and merged starting with the shortest-distance links, with each new addition checked for internal consistency and whether distances to other column members meet the column inclusion criterion.

The resulting sequence alignment is shown with Multalign Viewer and can be saved in various formats from that tool.

Calculation time scales approximately as N^3 ^with the number of models (Figure S1 [see supplementary.doc]).

### Matching distantly related proteins

Superpositions can be evaluated using the number of residues paired (N) and the corresponding root-mean-square deviation (RMSD). Since there is always a trade-off between higher N and lower RMSD, little can be said about a comparison where one superposition has both values higher or both lower than another. In fact, many N/RMSD pairs can describe the same superposition. For a given pair of structures, however, a superposition with both higher N and lower RMSD is clearly better.

Obtaining such data provides an example of how the sequence/structure tools in Chimera work together. Once the structures have been opened in Chimera, MatchMaker can be called from the menu or used via a command to superimpose them. The sequence alignment from MatchMaker could be shown with Multalign Viewer, but in the current situation, this would not be used. Information on the final (pruned) number of pairs and alpha-carbon RMSD is sent to the status line and Chimera Reply Log. As explained above, relatively few pairs may be used in MatchMaker's final fit, so Match -> Align would then be called from the menu to generate a full set of equivalences between the two structures, in the form of a sequence alignment. This sequence alignment is automatically shown with Multalign Viewer. Structure matching on all aligned positions would then be called from Multalign Viewer's menu to obtain an RMSD for the set of equivalent pairs. This procedure may sound elaborate, but actually takes just a few seconds.

Fischer and coworkers [14] collected a set of same-fold, low-identity protein pairs and rated their difficulty for sequence alignment and fold recognition. Table 1 shows N/RMSD data for the ten pairs rated most difficult. Superpositions were generated using MatchMaker with default alignment scoring, scoring without secondary structure, and scoring with only secondary structure, that is, 100% weighting of the secondary structure term.

**Figure 2:**
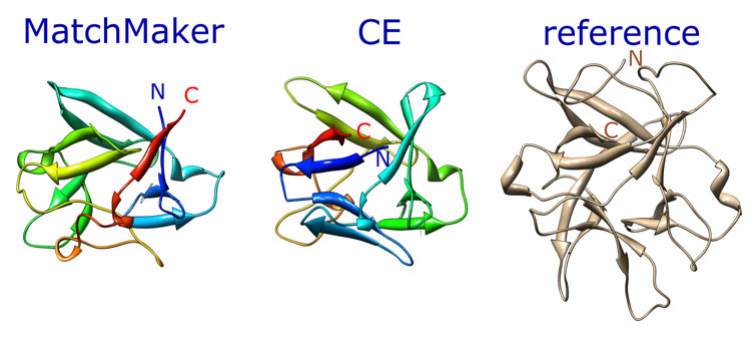
**Comparison of matched orientations (pair 8)**. Comparison of matched orientations from MatchMaker (default settings) and CE [26] (web server [34] with default settings). Each matched structure [PDB:4fgf] is rainbow-colored from blue at the N-terminus to red at the C-terminus. The reference structure [PDB:1tie] is shown in tan on the right.

Based on visual inspection and the results in Table 1, MatchMaker with default scoring superimposes nine of the ten pairs correctly. The fit of pair 8 appears close, but is likely wrong in that it implies a circular permutation of one protein relative to the other (shown in Figure 2 and discussed further in the Comparisons section). Without secondary structure scoring, none of the superpositions are correct; no result was obtained for pair 1 because iteration halted when the number of residue pairs fell below four, and the other nine pairs were superimposed incorrectly. With secondary structure scoring alone, the superpositions of seven pairs are grossly correct, but most of the fits are not as good as with default scoring. Pair 8 was also matched correctly, lacking the circular permutation obtained with default scoring, but pairs 4 and 7 were matched incorrectly.

Not surprisingly, secondary structure scoring makes MatchMaker sensitive to secondary structure assignments. We have found that recalculating secondary structure with the Kabsch and Sander algorithm [15] (as implemented within Chimera) instead of using pre-existing assignments yields very similar or improved results, depending on the pair (Table S1 [see supplementary.doc]). This option is on by default in MatchMaker. The improvement is likely due (at least in part) to the use of consistent criteria among the structures being matched. Secondary structure assignments in the input PDB files may have been generated with different criteria for different structures.

One might argue that the default matrix, BLOSUM-62, is not appropriate for such divergent proteins. To address this issue, analogous tests were performed with BLOSUM-30 instead (Table S2 [see supplementary.doc]). With secondary structure scoring (default weight of 30%), BLOSUM-30 superimposed all pairs correctly except pair 5. Thus, when combined with secondary structure scoring, BLOSUM-30 and BLOSUM-62 each correctly superimpose nine of the ten pairs. Without secondary structure scoring, BLOSUM-30 yielded only roughly correct matches for pairs 7 and 9, and incorrect matches for the others. This is better than the zero correct matches obtained with BLOSUM-62 alone, but still quite poor. Secondary structure scoring helps to generate better fits than can be obtained with sequence methods alone and decreases MatchMaker's sensitivity to the choice of substitution matrix.

Interestingly, end results are almost identical when the Smith-Waterman algorithm (local alignment) is used in MatchMaker instead of the default Needleman-Wunsch algorithm (global alignment) (Table S3 [see supplementary.doc]). Apparently, the pruning of pairs during iteration leads to use of the same or nearly the same set of positions in the final fit.

MatchMaker's purpose is to provide correct and useful superpositions for interactive study in a wide range of research situations (from closely related to broader groups of proteins, with varying amounts of sequence and structure data available), and to do so quickly and conveniently. It does not produce any significance metric and is not intended for remote homology detection. We envisioned it would be used on structures above the "twilight zone" of sequence identity, including trivial cases like different conformations of a given protein or mutants versus wild-type proteins. The ability to correctly match distantly related proteins is an added and somewhat unforeseen benefit.

### Comparisons with other programs

Several programs overlap in function with Chimera's Multalign Viewer. Alone or in combination with partner visualization programs, these generally allow adjustments to the sequence and structure displays and simple crosstalk between sequence and structure. Some of the programs allow alignment editing and superposition of structures based on the sequence alignment. ModView [16] and its stand-alone successor Friend [17] also integrate phylogenetic information such as dendrograms. ViTO [18] displays alignment insertions and deletions on structures and reports threading energies. STRAP [19] is designed to handle large numbers of sequences; structure visualization is provided by a partner program such as PyMOL [20] or Visual Molecular Dynamics (VMD) [21]. ModView [16] and STRAP [19] can be used as Web plug-ins. Cn3D [22] is a web plug-in distributed as part of Entrez [23]. STING [24,25] is a web plug-in that can display pairwise alignments from Combinatorial Extension [26] (CE, discussed further below); many types of structure-related data are shown on the sequence alignment, and Jmol [27] is used to display the structures.

Multalign Viewer cannot be used as a Web plug-in. Other disadvantages, which we plan to address in the future, are that editing capabilities are limited and there is no interaction with phylogenetic information such as dendrograms. Although there is no hard limit on the number of sequences that can be handled, Chimera uses more memory per sequence and structure than many other programs. Advantages of Multalign Viewer include the ability to add arbitrary sequence annotations, automatic propagation of sequence annotations to associated structures, sophisticated options for calculating conservation, and the ability to wrap alignment text instead of presenting it as a horizontal bar.

It is beyond the scope of this paper to discuss the many existing methods for protein superposition. For comparison with MatchMaker, we will mention a few that are integrated with programs with broader sequence/structure visualization capabilities. The align command in PyMOL [20] is similar to MatchMaker without the secondary structure term. It generates a sequence alignment and fits structures accordingly, with or without fit iteration. The Multiple Alignment plug-in [28] to VMD [21] uses STAMP [29]. STAMP starts with approximate matches that can be generated by sequence methods or by scanning segments of a protein against the others for structural similarity. It then refines the fits and determines a sensible order in which to add successive structures to a multiple superposition. Cn3D [22] aligns structures using VAST (Vector Alignment Search Tool) [30], which pairs secondary structure elements of similar types, orientations, and connectivities. The program Friend [17] can superimpose structures with TOPOFIT [31] or Combinatorial Extension (CE) [26]. STRAP [19] can use CE [26] or an unpublished method by Goede. TOPOFIT, CE, and the Goede method are quite different from each other, but they all use structure information, not sequence (although residue identity can be used in the optimization stage of CE).

To our knowledge, only MatchMaker can use both sequence and structure information in the initial round of matching. Methods that use only sequence information often fail when proteins are highly divergent. Conversely, methods that use only structure are discarding any signal present in the sequences.

In practice, superpositions of low-identity pairs from Chimera are comparable to those from structure-based methods. Table 2 shows results obtained with Chimera, TOPOFIT [31], and CE [26] for the same pairs of structures as in Table 1. In terms of N and RMSD, the Chimera results fall between those of TOPOFIT and CE for all pairs except pair 8.

The agreement between Chimera's MatchMaker and CE is more evident when a consistent method is used to obtain equivalences. Columns Chimera and CE/MA in Table 2 show data for the equivalences determined by Match -> Align with cutoff 5.0 angstroms. Only pairs 8 and 10 show major differences. As mentioned above, the superposition of pair 8 from MatchMaker implies a circular permutation of one protein relative to the other (Figure 2). Allowing for circular permutation when using Match -> Align on this superposition yields 105 pairs matched with 2.3 angstroms RMSD. The non-permuted alignment from Match -> Align for the CE superposition, however, also yields 105 pairs but with a lower RMSD (Table 2), further evidence that the CE superposition is the correct one. The opposite is true for pair 10: in the CE superposition, a central sheet is shifted over by one strand relative to the correct match. Importantly, default settings were used in Chimera; it is likely that performance could be improved with pair-specific parameter tuning or with additional cycles of matching, pruning, and creating another sequence alignment.

The ratio of sequence to structure information used by MatchMaker is adjustable; the ratio most appropriate for a given problem depends on the divergence of the proteins and the intended use of the results. However, results are generally robust to a wide range of parameter settings, and there should be little need for hunting down an optimal set of parameters. The combined sequence-structure score is similar in spirit to that used in STACCATO [32] for a different purpose (see below). Disadvantages of MatchMaker are that it is inherently pairwise and does not provide guidance on which structure should be used as the reference for overlaying multiple structures.

Few programs exist to derive a sequence alignment from an arbitrary pre-existing superposition. The only one we know of besides Match -> Align is STACCATO [32]. The methods are very different, however. STACCATO uses a unique sequence-structure score with contributions from residue similarity, residue secondary structure environment, and spatial proximity. In Chimera, the first two types of information are used by MatchMaker to fit the structures, but only the third, spatial proximity, is used by Match -> Align to determine equivalences. STACCATO can use a distance cutoff with an "all others" column inclusion criterion, but it can also operate without a cutoff; the score will still favor the alignment in sequence of residues close in space.

The separation of structure matching from generation of a structure-based sequence alignment is important when a user has already obtained the desired fit using protein-specific knowledge, such as by matching a constellation of important active site residues, or by fitting using only the atoms of a bound ligand or cofactor. In such cases, it would defeat the purpose to use a global structure-based superposition program to produce a sequence alignment, as that would simultaneously alter the structural fit. Separation of these two tasks also makes it more obvious that many different sequence alignments are consistent with a given structural superposition; users can vary the column inclusion criterion and cutoff distance as appropriate for the intended purpose of the alignment and the divergence of the proteins under study.

The Chimera sequence/structure tools provide access to many parameter settings via graphical interfaces. A further advantage, apart from their individual merits, is the convenience of having the tools work together within a single program. Advantages relating to Chimera as a whole are a broad feature set for structure analysis, detailed and searchable documentation, program extensibility, and continuing development. Finally, although Chimera has many features, it is not meant to be a closed system; it can read many common file formats. This allows researchers to use superpositions or sequence alignments from outside programs or databases in lieu of one of Chimera's similar tools, while still benefiting from features of the other tools discussed.

## Future directions

We have many ideas for future developments; some of the more concrete and near-term issues are mentioned here.

Editing in Multalign Viewer is currently limited to shifting highlighted blocks of sequence to create, extend, or remove gaps; residues cannot be changed, and they can only be deleted by rewriting a new file minus those residues. We plan to allow entire sequences and alignment columns to be deleted without new file creation.

We envision reading phylogenetic data along with an alignment and displaying the tree and sequences side by side. Nodes in the alignment would be collapsible to representative or consensus sequences, and analyses such as conservation calculations could be applied to only the members of a node.

Although several ways to calculate conservation are already included and users can create their own header information by loading a text file, we plan to make headers even more powerful by allowing users to define their own header functions. While loading a header file places static values in the header line, values produced by a function can be recomputed for different alignments or as the alignment is changed by editing. We will also allow headers to be graphical representations, such as cylinders and thick arrows to symbolize stretches of helix and strand. Another option will be to show residue numbers at the ends of lines.

In principle, the sequence-structure tools in Chimera can be used on nucleic acids, but little attention has been paid to this application. MatchMaker has a DNA matrix, but it would be helpful to add a matrix that works with RNA.

Chimera as a whole will benefit from ongoing efforts to improve its speed and memory usage.

## Conclusion

Chimera includes tools for integrating protein sequence and structure information. The Multalign Viewer tool displays internally and externally generated sequence alignments with customizable layout and coloring. Structures opened in Chimera are automatically associated with sequences in the alignment, as appropriate. Association enables matching of structures according to the sequence alignment, display of alignment-related data such as conservation on the structures, and display of structure-related data on the sequence alignment. In the absence of a pre-existing alignment, the MatchMaker tool constructs a new alignment and matches structures accordingly. The alignment score includes secondary structure information, extending the usefulness of MatchMaker to distantly related proteins. The Match -> Align tool creates sequence alignments of two or more proteins that have already been superimposed, using only the spatial proximities of their alpha-carbons.

Advantages of using Chimera for sequence/structure research include a rich set of features co-existing in a single program, certain unique methods, facile integration of user data, access to the broader visualization and analysis capabilities of Chimera, program extensibility, detailed documentation, and continuing development.

## Availability and requirements

Project name: UCSF Chimera

Project home page: 

Operating systems: Microsoft Windows, Linux, Apple Mac OS X, SGI IRIX, and HP Tru64 Unix

Programming language: Python, C++

License: Chimera is free to academic and non-profit users, subject to an online license agreement. Commercial use requires a fee and a separate, written license agreement (interested parties should contact tef@cgl.ucsf.edu). The distribution bundle includes user documentation, executables, and all Python code. The full source code including C++ files is also available for download.

## Abbreviations

UCSF: University of California San Francisco; GUI: graphical user interface; GCG MSF: Genetics Computing Group Multiple Sequence Format; GCG RSF: Genetics Computing Group Rich Sequence Format; NBRF: National Biomedical Research Foundation; PIR: Protein Information Resource; PDB: Protein Data Bank; SCOP: Structural Classification of Proteins; RMSD: root-mean-square deviation; VMD: Visual Molecular Dynamics; STAMP: STructural Alignment of Multiple Proteins; VAST: Vector Alignment Search Tool; CE: Combinatorial Extension; STACCATO: STructural sequence Alignment, Correspondence and Conservation Analysis TOol.

## Authors' contributions

EFP designed and implemented the Chimera extensions described in this work, with major input from ECM. ECM wrote the user documentation, generated the results presented herein, and wrote the paper, with major input from EFP. Along with EFP, GSC, CCH, and TEF designed and implemented the Chimera system. All authors have participated in discussions about this manuscript and the tools described.

## Supplementary Material

Additional File 1 Supplementary data. Default parameters in Chimera version 1.2199, MatchMaker results with different parameter settings (Tables S1–S3), scaling of Match -> Align computation time (Figure S1). Click here for file

## Figures and Tables

**Table 1:** Chimera results for difficult structure pairs

pair	PDB ID	PDB ID	MM-default	MM-noSS	MM-allSS
1	3chy	2fox	21/1.2, 88/2.2	NR	22/1.1, 88/2.4
2	2aza A	1paz	34/1.2, 79/2.0	8/1.5, 19/2.7	16/1.4, 74/2.3
3	1cew I	1mol A	29/1.0, 73/1.9	6/0.8, 7/1.5	26/0.9, 70/2.0
4	1cid	2rhe	38/1.1, 90/2.0	10/1.3, 21/2.4	11/1.3, 57/2.5
5	1crl	1ede	32/1.3, 184/2.5	6/1.3, 26/3.2	13/1.3, 166/2.6
6	2sim	1nsb A	33/1.2, 257/2.6	4/1.5, 28/3.2	20/1.2, 174/2.9
7	1ten	3hhr B	53/1.1, 82/1.3	4/1.0, 16/2.9	5/0.9, 22/2.9
8	1tie	4fgf	21/1.3, 73/2.2	6/1.1, 22/2.7	15/1.2, 97/2.2
9	2snv	5ptp	32/1.3, 118/2.3	8/1.5, 22/2.4	12/1.4, 103/2.4
10	1gp1 A	2trx A	33/1.0, 89/1.8	5/0.5, 17/2.7	30/1.2, 88/1.7

Results are of the form N/RMSD, where N is the number of residue pairs matched and RMSD is the corresponding alpha-carbon root-mean-square deviation. The first set per column represents the final MatchMaker iteration (after pruning), while the second represents all equivalences from subsequent use of Match -> Align (cutoff 5.0 angstroms). **MM-default**: MatchMaker with default parameters (BLOSUM-62, 30% secondary structure weighting, prior secondary structure calculation, iteration cutoff 2.0 angstroms; further details are available in the supplementary information [see supplementary.doc]). **MM-noSS**: same as MM-default except without secondary structure scoring. **MM-allSS**: same as MM-default except with only the secondary structure term (100% weighting). NR: no results; MatchMaker iteration ended prematurely when the number of residue pairs fell below four.

**Table 2:** Results from different programs for difficult structure pairs

pair	PDB ID	PDB ID	Chimera	TOPOFIT	CE	CE/MA
1	3chy	2fox	88/2.2	19/0.7	108/3.6	93/2.6
2	2aza A	1paz	79/2.0	72/1.7	84/2.9	81/2.4
3	1cew I	1mol A	73/1.9	73/1.6	81/2.3	78/1.9
4	1cid	2rhe	90/2.0	69/1.5	97/2.9	88/1.9
5	1crl	1ede	184/2.5	143/1.9	219/3.8	183/2.3
6	2sim	1nsb A	257/2.6	207/2.0	275/3.0	266/2.5
7	1ten	3hhr B	82/1.3	81/1.4	87/1.9	85/1.6
8	1tie	4fgf	73/2.2	88/1.6	116/2.9	105/2.1
9	2snv	5ptp	118/2.3	83/1.8	130/3.1	118/2.4
10	1gp1 A	2trx A	89/1.8	96/1.6	64/5.2	54/2.7

Results are of the form N/RMSD, where N is the number of residue pairs matched and RMSD is the corresponding alpha-carbon root-mean-square deviation. Chimera: results from using MatchMaker and then Match -> Align with default settings, as reported under MM-default in Table 1. TOPOFIT [31] results were obtained using the web server [35]. CE [26] results were obtained using the web server [34] with default settings. CE/MA: equivalences obtained by using Match -> Align with cutoff 5.0 angstroms on the superpositions from CE.
