# Supplementary material for: Tools for integrated sequence-structure analysis with UCSF Chimera
Source: BMC Bioinformatics. 2006 Jul 12;7:339. doi: 10.1186/1471-2105-7-339 (PMC1570152; doi:10.1186/1471-2105-7-339)
Supplement: Additional File 1 — Supplementary data. Default parameters in Chimera version 1.2199, MatchMaker results with different parameter settings (Tables S1–S3), scaling of Match -> Align computation time (Figure S1). [file 1471-2105-7-339-S1.doc]

# Tools for integrated sequence-structure analysis with UCSF Chimera

# Supplementary data

## Default parameters in Chimera version 1.2199.

Default sequence alignment parameters in MatchMaker are to use the Needleman-Wunsch algorithm, 70% weighting of amino acid similarity (BLOSUM-62 matrix), 30% secondary structure weighting (scoring matrix elements helix-helix 6, strand-strand 6, other-other 4, helix-strand –9, helix-other –6, strand-other –6), and prior secondary structure calculation. The default gap extension penalty is 1. When secondary structure scoring is used, the default penalty for opening a gap is 18 within a helix or strand and 6 otherwise; when secondary structure scoring is not used, the default penalty for opening a gap is 12. Structural fits are iteratively improved with a cutoff of 2.0 angstroms.

## Table S1 - MatchMaker without secondary structure recalculation

| pair | PDB ID | PDB ID | MM-default | MM-noksdssp |
| --- | --- | --- | --- | --- |
| 1 | 3chy | 2fox | 21/1.2, 88/2.2 | 27/1.0, 80/2.3 |
| 2 | 2aza A | 1paz | 34/1.2, 79/2.0 | 31/1.3, 78/2.0 |
| 3 | 1cew I | 1mol A | 29/1.0, 73/1.9 | 23/0.7, 76/1.7 |
| 4 | 1cid | 2rhe | 38/1.1, 90/2.0 | NR |
| 5 | 1crl | 1ede | 32/1.3, 184/2.5 | 21/1.4, 147/2.8 |
| 6 | 2sim | 1nsb A | 33/1.2, 257/2.6 | 47/1.2, 259/2.4 |
| 7 | 1ten | 3hhr B | 53/1.1, 82/1.3 | 41/1.0, 70/1.8 |
| 8 | 1tie | 4fgf | 21/1.3, 73/2.2 | 24/1.2, 71/2.2 |
| 9 | 2snv | 5ptp | 32/1.3, 118/2.3 | 15/1.2, 82/2.7 |
| 10 | 1gp1 A | 2trx A | 33/1.0, 89/1.8 | 30/0.9, 90/1.8 |

Results are of the form N/RMSD, where N is the number of residue pairs matched and RMSD is the corresponding alpha-carbon root-mean-square deviation. The first set per column represents the final MatchMaker iteration, while the second represents all equivalences from subsequent use of Match ->Align (cutoff 5.0 angstroms). **MM-default**: MatchMaker with default parameters. **MM-noksdssp**: same as MM-default except without recalculation of secondary structure in proteins with existing assignments. Note the calculation is still performed for PDB entries without secondary structure records [PDB:1cew, PDB:1cid, PDB:2sim, PDB:1ten]. NR: no results; MatchMaker iteration ended prematurely when the number of residue pairs fell below four.

## Table S2 - MatchMaker with BLOSUM-30 instead of BLOSUM-62

| pair | PDB ID | PDB ID | MM-B30 | MM-B30noSS |
| --- | --- | --- | --- | --- |
| 1 | 3chy | 2fox | 23/1.2, 87/2.5 | 8/1.4, 41/2.6 |
| 2 | 2aza A | 1paz | 25/1.3, 77/2.1 | 20/1.1, 48/2.4 |
| 3 | 1cew I | 1mol A | 17/1.0, 70/2.2 | 8/1.5, 24/2.4 |
| 4 | 1cid | 2rhe | 46/1.1, 91/2.1 | 9/1.5, 31/2.7 |
| 5 | 1crl | 1ede | 5/0.9, 69/2.9 | 4/1.0, 57/2.8 |
| 6 | 2sim | 1nsb A | 38/1.2, 257/2.5 | 9/1.4, 100/3.3 |
| 7 | 1ten | 3hhr B | 50/1.1, 83/1.4 | 13/1.0, 84/1.6 |
| 8 | 1tie | 4fgf | 15/1.3, 89/2.6 | 5/1.6, 31/3.1 |
| 9 | 2snv | 5ptp | 23/1.3, 120/2.3 | 10/1.3, 77/2.3 |
| 10 | 1gp1 A | 2trx A | 33/1.0, 89/1.8 | 4/1.5, 23/2.6 |

Results are of the form N/RMSD, where N is the number of residue pairs matched and RMSD is the corresponding alpha-carbon root-mean-square deviation. The first set per column represents the final MatchMaker iteration, while the second represents all equivalences from subsequent use of Match ->Align (cutoff 5.0 angstroms). **MM-B30**: MatchMaker with default parameters except using BLOSUM-30 instead of BLOSUM-62. **MM-B30noSS**: same as MM-B30 except without secondary structure scoring.

## Table S3 - MatchMaker using local instead of global alignment

| pair | PDB ID | PDB ID | MM-SW | MM-B30SW |
| --- | --- | --- | --- | --- |
| 1 | 3chy | 2fox | 21/1.2, 88/2.2 | 23/1.2, 87/2.5 |
| 2 | 2aza A | 1paz | 34/1.2, 79/2.0 | 26/1.3, 77/2.1 |
| 3 | 1cew I | 1mol A | 16/1.0, 75/1.7 | 14/1.1, 63/2.4 |
| 4 | 1cid | 2rhe | 38/1.1, 90/2.0 | 46/1.1, 91/2.1 |
| 5 | 1crl | 1ede | 32/1.3, 184/2.5 | 21/1.1, 127/2.9 |
| 6 | 2sim | 1nsb A | 33/1.2, 257/2.6 | 39/1.3, 258/2.5 |
| 7 | 1ten | 3hhr B | 47/1.0, 82/1.3 | 50/1.1, 83/1.4 |
| 8 | 1tie | 4fgf | 22/1.3, 86/2.4 | 17/1.0, 99/2.1 |
| 9 | 2snv | 5ptp | 19/0.9, 120/2.4 | 30/1.1, 118/2.3 |
| 10 | 1gp1 A | 2trx A | 34/1.0, 89/1.8 | 33/1.0, 89/1.8 |

Results are of the form N/RMSD, where N is the number of residue pairs matched and RMSD is the corresponding alpha-carbon root-mean-square deviation. The first set per column represents the final MatchMaker iteration, while the second represents all equivalences from subsequent use of Match ->Align (cutoff 5.0 angstroms). **MM-SW**: MatchMaker with default parameters except using the Smith-Waterman algorithm instead of Needleman-Wunsch. **MM-B30SW**: same as MM-SW except also using BLOSUM-30 instead of BLOSUM-62.

## Scaling of Match -> Align computation time.

Calculation times depend on several interacting factors, including the specific structures and how closely they match, but the tendency is for increasing time with increasing numbers of structures, longer sequences, larger cutoff distances, and a more lenient column inclusion criterion (“at least one other” instead of “all others”). As an example, Figure S1 shows timings for structures in the HOMSTRAD [1] amino acid dehydrogenase family.

**
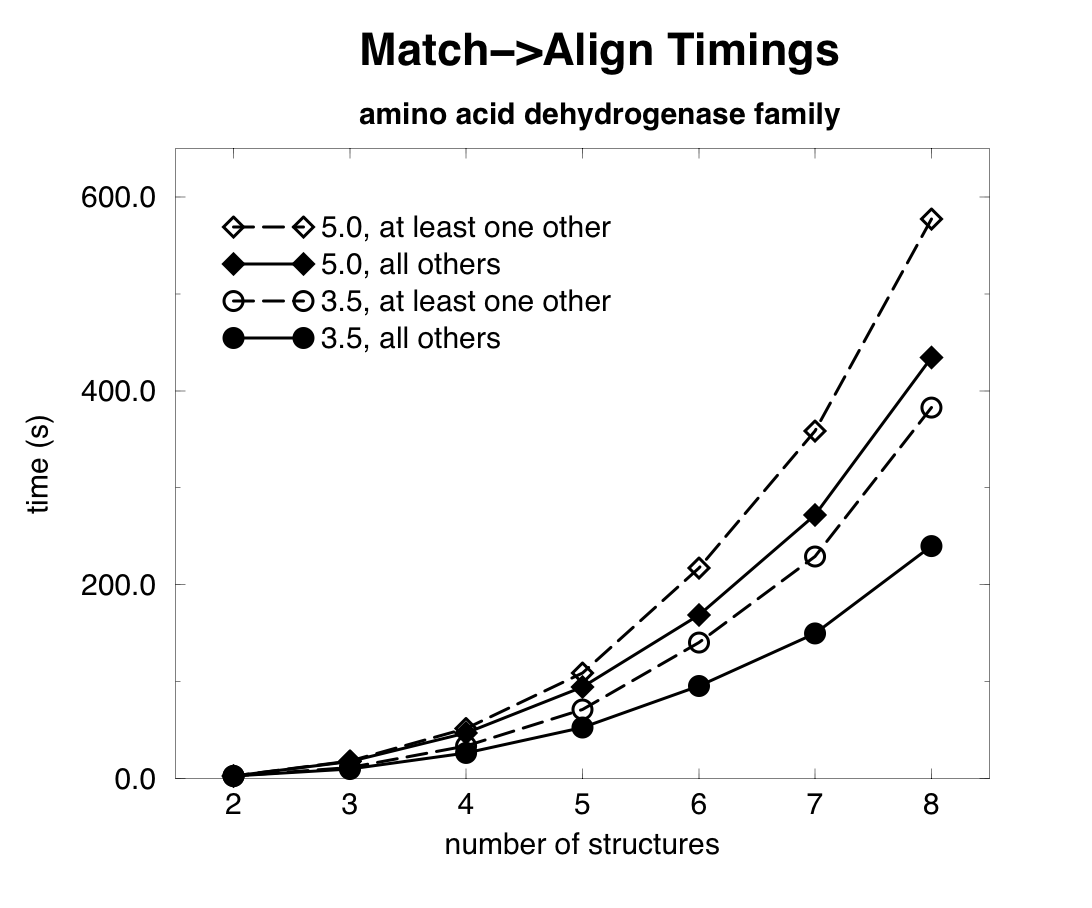
**

**Figure S1**

Wall clock times for generating a multiple sequence alignment from a superposition with Match -> Align (Mac OS X with 2.7 GHz PowerPC G5 processor and 1 GB memory; Chimera version 1.2199). The HOMSTRAD [1] amino acid dehydrogenase family includes eight chains, with average length 415 residues [PDB:1c1d chain A, PDB:1leh chain A, PDB:1b26 chain A, PDB:1hwx chain A, PDB:1gtm chain A, PDB:1bgv chain A, PDB:1euz chain A, PDB:1bvu chain A]. These chains were superimposed using MatchMaker with default settings and 1c1d as the reference. For the Match -> Align calculations, the set of seven was obtained by leaving out 1bvu, the set of six by also leaving out 1euz, etc., in reverse order of the listing above. The four sets of runs differ in cutoff distance to other residues in the column (diamonds, 5.0 angstroms; circles, 3.5 angstroms) and/or column inclusion criterion (open symbols, to at least one other residue; filled symbols, to all other residues).

**References**

1) Stebbings LA, Mizuguchi K: **HOMSTRAD: recent developments of the Homologous Protein Structure Alignment Database.** *Nucleic Acids Res* 2004, **32:**D203-207
